# Evaluation of methods to detect in vitro biofilm formation by staphylococcal clinical isolates

**DOI:** 10.1186/s13104-018-3820-9

**Published:** 2018-10-10

**Authors:** Sarita Manandhar, Anjana Singh, Ajit Varma, Shanti Pandey, Neeraj Shrivastava

**Affiliations:** 1 Tri-Chandra Multiple College, Tribhuvan University, Kathmandu, Nepal; 2 Amity Institute of Microbial Technology, Amity University Uttar Pradesh, Noida, UP 201303 India; 3 Central Department of Microbiology, Tribhuvan University, Kathmandu, Nepal; 4 The University of Southern Mississippi, Hattiesburg, MS 39406 USA; 5 Institute of Biotechnology, Zhejiang University, Hangzhou, 310058 People’s Republic of China

**Keywords:** *Staphylococcus* spp., Biofilm, Clinical specimens, Phenotypic assays, Genotypic assay

## Abstract

**Objective:**

Staphylococcus genus comprising both *Staphylococcus aureus* and coagulase negative staphylococci (CoNS) are widely distributed in nature and can infect diversity of hosts. Indeed, staphylococci are the major pathogens causing biofilm associated infections caused by contaminated hospital indwelling devices. These infections are persistent in nature being highly refractory to various stresses including antibiotics. Implementation of efficient diagnostic techniques for the biofilm production would help minimize the disease burden. Thus, early detection of pathogenic strains producing biofilms warrant the utmost importance in diagnostic laboratories especially in resource limited settings.

**Result:**

Among 375 isolates collected from different clinical specimens, 214 (57%) were identified as coagulase negative staphylococci and 161 (43%) *S. aureus.* Detection of *In*-*vitro* biofilm formation in these isolates were carried out by three commonly used phenotypic assays and a genotypic assay. While evaluating the results, tissue-culture method with supplemented glucose and sucrose showed the best correlation with the results of genotypic assay.

**Electronic supplementary material:**

The online version of this article (10.1186/s13104-018-3820-9) contains supplementary material, which is available to authorized users.

## Introduction

*Staphylococcus* spp., widely distributed in nature, colonize the skin and anterior nares of humans. However, upon achieving the favorable environment, they can infect the diversified hosts [1, 2] due to the presence of numerous virulence factors including exotoxins, enzymes, surface proteins, ability of biofilm production and acquisition of resistance to multiple drugs [3–5].

Biofilm is a structured community of bacterial cells enclosed in self-produced polymeric matrix adherent to an inert or living surface [6–8]. As implant devices are increasingly used in medical practice, staphylococcal infections are now considered one of the major nosocomial infections [9, 10]. Biofilm associated infections are characteristically refractory to different stresses including host immune defense and antibiotics, leading to persistent infections [2, 11–13].

The polysaccharide intercellular adhesin (PIA) is the main biomolecule responsible for cell aggregation and biofilm formation. PIA biosynthesis is carried out by the proteins encoded by the *ica* operon *(icaADBC)* [14–16]. Given that staphylococcal infections associated with medical devices have significant impact on morbidity, mortality and socio-economic burden, prevention and management of such infections remains a priority. Thus, detection and differentiation of staphylococci in their ability to form biofilm in routine laboratory practice bear great importance to initiate effective treatment measures and minimize unsuccessful antibiotic therapies [7, 17].

Various phenotypic methods like Congo-red agar method (CRA), tube method (TM), tissue culture plate method (TCP), electron microscopy, confocal scanning microscopy and bioluminescent assay are available for the detection of biofilm formation in staphylococcal infections [7, 18]. Detection of biofilm related genes using PCR techniques have been increasingly used, but this may be infeasible as routine diagnostic in a resource-limited country like Nepal. Therefore, in the present study, we sought to compare and evaluate the sensitivity and specificity of three most commonly used phenotypic assays with the genotypic assay to detect biofilm production.

## Main text

### Materials and methods

A total of 375 clinical staphylococcal isolates were collected from two tertiary care hospitals from 2015 to 2017. Staphylococci were isolated and identified from various clinical samples by standard microbiological techniques [19]. High biofilm producer strain *Staphylococcus epidermidis* ATCC 35984 was used as reference strain in all the tests performed. All experiments were performed in triplicate and repeated thrice.

### Screening of biofilm production

#### Phenotypic assay

The in vitro biofilm production was measured using phenotypic assays CRA, TM and TCP methods. In CRA method, biofilm production was measured qualitatively described [20]. The black colonies with dark consistency were regarded as strong biofilm producers while the pink colonies as biofilm non-producers.

TM, a qualitative method for the detection of biofilm formation was performed as described [21]. Briefly, biofilm formation was considered positive when a visible film was observed along the inner wall and bottom of tube. Depending on this, isolates were scored as 0, 1, 2 and 3 for absence, weak, moderate and strong biofilm formation respectively.

TCP, a quantitative method was used as described by Christensen et al. with slight modification [21], using trypticase soy broth (TSB), TSB with 1% glucose and Brain Heart Infusion (BHI) broth with 2% sucrose. Optical densities (OD) of both the dry plates and eluted stain was measured using micro ELISA auto reader at OD 630 nm. Mean OD value < 0.120, 0.120–0.240 and > 0.240 were classified as non/weak, moderate and strong biofilm adherence respectively [18].

#### Genotypic assay

Polymerase chain reaction (PCR) was used to detect *icaA* and *icaD* genes. The genomic DNA was extracted using a DNA extraction kit following the manufacturer instructions (Thermo Fischer). The forward and reverse primers (Solis Biodyne, Denmark) for *icaA* used were 5′-TCTCTTGCAGGAGCAATCAA and 5′-TCAGGCACTAACATCCAGCA respectively. For *icaD*, 5′-ATGGTCAAGCCCAGACAGAG as forward and 5′-CGTGTTTTCAACATTTAATGCAA as reverse primer. The PCR product was analyzed in 2% agarose gel stained with SYBR safe (Invitrogen) dye [22].

### Statistical analysis

Sensitivity and specificity were evaluated by comparing the result of phenotypic methods with genotypic methods as standard. Different phenotypic methods were also compared with TCP as standard for phenotypic assays. Chi square test was used to evaluate the apparent differences for significance at 95% confidence level using IBM SPSS v 21.0.

### Results

Based on coagulase test, we differentiated 375 isolates into 214 (57%) CoNS and 161 (43%) *S. aureus.* Among six CoNS species identified, *S. epidermidis* was the most prevalent (57.5%), followed by *S. saprophyticus* (18.7%), *S. haemolyticus* (11.2%), *S. hominis* (7.0%), *S. capitis* (5.6%) (Additional file 1: Table S1).

Among 375 isolates, 86 (22.9%) isolates were found to possess both *icaA* and *icaD* genes comprising 45 (28%) *S. aureus* and 41 (19.2%) CoNS which predominantly constituted 29 (33.7%) *S. epidermidis* isolates (Table 1).

**Table 1:** Screening of in vitro biofilm production with different methods

Biofilm production	CRA	TM	TCP	*Ica* genes
High	20 (5.3%)	63 (16.8%)	21 (5.6%)	86 (22.9%)
Moderate	26 (6.9%)	66 (17.6%)	91 (24.3%)	–
Weak/non	329 (87.7%)	246 (65.6%)	263 (70.1%)	289 (77.1%)
P value	0.390	0.000	0.374	

*CRA* Congo Red Agar Method, *TM* Tube Method, *TCP* Tissue Culture Plate Method

Among all isolates, 20 (5.3%) isolates were positive in CRA while 329 (87.7%) isolates were biofilm non-producers with red colonies. It was found that detection of biofilm production by TM method was statistically significant when compared with presence of *ica* genes whereas CRA and TCP methods were statistically insignificant (Table 1) (Additional file 2: Table S2). We observed 14% sensitivity and 88% specificity while comparing CRA method with the genotypic assay. This shows no good correlation of CRA method with genotypic assay (Table 3).

In TM method, 63 (16.8%) isolates were found to be strong, 66 (17.6%) moderate, and 246 (65.6%) biofilm non-producers. The strong biofilm producers included 19 (11.8%) *S. aureus* and 44 (20.6%) CoNS species with highest frequency in *S. epidermidis* 33 (25.6%). The sensitivity and specificity of the tube method showed 64% and 74% respectively to genotypic assay (Tables 1, 3).

The TCP method was used to assess biofilm production using three variations in media. In TCP with TSB only, 21 (5.6%) isolates with 4 (2.5%) *S. aureus* and 17 (7.9%) CoNS showed strong biofilm production. An addition of 1% glucose to TSB medium increased biofilm detection in 83 (22.1%) comprising 48 (19.8%) *S. aureus* and 35 (16.4%) CoNS species. In BHI, incorporated with 2% sucrose also increased biofilm detection including 41 (25.5%) *S. aureus* and 66 (30.8%) CoNS species. Our study showed the induction of biofilm production on addition of nutrients specially glucose and sucrose. When TCP was compared with the genotypic assay, among 83 strong biofilm producers, 20 (24.1%) were shown to possess *icaAD* genes. Our result showed no significant difference in biofilm production between dry plate and ethanol-eluted TCP method (Tables 2, 3; Additional file 3: Table S3).

**Table 2:** Frequency of biofilm production in TCP method with different media composition

Biofilm formation	TSB only		TSB + glucose		BHI + sucrose	
	Dry	Elution with ethanol	Dry	Elution with ethanol	Dry	Elution with ethanol
High	21 (5.6%)	22 (5.9%)	83 (22.1%)	87 (23.2%)	107 (28.5%)	97 (25.9%)
Moderate	91 (24.3%)	85 (22.7%)	91 (24.3%)	109 (29.1%)	122 (32.5%)	132 (35.2%)
Weak/non	263 (70.1%)	268 (71.5%)	201 (53.6%)	179 (47.7%)	146 (38.9%)	146 (38.9%)

TSB, Tryptic Soy Broth; BHI, Brain Heart Infusion

**Table 3:** Statistical evaluation of phenotypic methods compared with genotypic method

Screening methods	Sensitivity	Specificity	Positive predictive value	Negative predictive value	Accuracy
CRA	14	88.2	26.1	77.5	71.2
TM	64	74.4	42.6	87.8	72
TCP-dry	33.7	71.3	26	78.3	62.7
TCP-elution	30.2	72	24.3	77.6	62.4

The biofilm production is accurately confirmed by detecting the genes involved in biofilm formation. But PCR technique as routine diagnosis is impractical in resource-limited countries like Nepal. In this scenario, implementation of easier and reliable phenotypic method would be more appropriate. Therefore, we sought to evaluate CRA, TM, and modified TCP method with standard TCP method. The results revealed the CRA method with the highest specificity (86%) but the lowest sensitivity (8%). With that, the modified TCP method using BHI with 2% sucrose was 80% sensitive with 57% accuracy rate for differentiating biofilm producers and non-producers. Addition of glucose in TSB corresponded to sensitivity and specificity of 59% when compared with the TCP method. These results suggested that modified TCP method using BHI with 2% sucrose and/or TSB supplemented with glucose, to be more reliable than those without supplements for detecting staphylococcal biofilm production (Additional file 4: Table S4).

### Discussion

For high disease burden of biofilm associated staphylococcal infections, a reliable and prompt diagnostic method is essential in health care facilities [2, 23]. Therefore, in this study, we evaluated three phenotypic, and a genotypic method of in vitro biofilm detection. To the best of our knowledge, this is first study using genotypic assay to detect in vitro biofilm production in clinical samples in Nepal.

In this study, 375 clinical staphylococcal isolates retained from various specimens were identified as *S. aureus* and CoNS in 161 (43%) and 214 (57%) isolates respectively. Consistent with previous studies, [24], *S. epidermidis* was the predominant CoNS species corresponding to 123 (57.5%) isolates. Because of its adaptive ability and highest dominance on human skin and mucosa [25], *S. epidermidis* has been reported the most prevalent in multiple studies [26, 27].

A plethora of studies demonstrate the causal link between staphylococcal biofilm and the presence of *ica* operon [3, 28–33], which in turn are involved in the PIA production; the most extensively characterized staphylococcal biofilm component [7, 29, 34–36]. In the present study, concomitant presence of *icaA* and *icaD* genes was detected in 86 (22.9%) staphylococcal isolates. Among CoNS, 29 (34%) *S. epidermidis* isolates found to possess *icaAD* genes. Los et al. showed the prevalence of *ica* operon in 27.4% nasopharyngeal *S. epidermidis* isolates from hospitalized patients [37]. Oliviera et al. detected *ica* genes in 40% CoNS isolated from clinical specimen and nares of healthy individuals [7]. Likewise, Cafiso et al., Nasr et al. and deSilva et al. showed 37%, 32% and 40% staphylococcal isolates positive for *ica* genes respectively [31, 34, 38].

CRA method showed slime production in 46 (12.2%) staphylococcal isolates. The sensitivity and specificity of CRA method was only 14% and 88% respectively as compared to genotypic assay. Arciola and colleagues also identified eight and six CRA negative isolates possessing *ica* genes in two consecutive studies [16, 39]. Similarly, Cafiso et al. and Fitzpatrick et al. also showed the reduced accuracy of this method to biofilm production [34, 40]. All these evidences suggest that, despite being easier and faster, CRA method cannot be relied upon for precise detection of biofilm producers in routine diagnostic laboratory.

TM showed 63 (16.8%) isolates as strong, 66 (17.6%) moderate and 246 (65.6%) weak/non-biofilm producers. The TM results showed 64% sensitivity and 74% specificity as compared to the genotypic assay. Consistence with the previous study [7], TM among phenotypic assays in our study demonstrated the best correlation with genotype assay.

The expression of *ica* genes in vitro studies have been reported to be highly variable depending on the composition of media as their expression is induced by the stresses with additional sugars [18, 41]. In only TSB, 112 (30%) isolates produced biofilm, while adding 1% glucose, the number of biofilm positive isolates increased to 174 (46.4%). This is consistent with the previous studies showing less positive results in TSB only medium [18, 42]. Furthermore, the biofilm formation in BHI agar with 2% sucrose drastically increased number of biofilm producers to 229 (61%). When the presence of *icaAD* genes was compared with TCP method, sensitivity increased on adding 1% glucose and 2% sucrose as compared to TSB only. These evidences suggest that biofilm formation by staphylococci depends on growth conditions. Indeed, the use of sugar as supplement in the media was found to be essential for biofilm formation [7, 18]. The use of additional sugar amount in a medium produces a stress condition that stimulates the fermentation reaction, resulting anaerobic condition that favors the production of PIA and consequently increasing biofilm production [8, 43]. Taken together, these results indicate that the expression of *ica* gene is highly variable and induced by many factors including incorporation of sugar, salt, ethanol in the culture media [6–8, 18, 40, 44].

Detection of *ica* genes by PCR method has been demonstrated to be highly reliable to detect biofilm formation [3, 7, 29]. However, previous studies have shown evidences that presence of *ica* gene doesn’t always correlate with biofilm production. For example; the study by deSilva demonstrated that only 59% of *ica* positive *S. epidermidis* isolates were found to be positive in CRA method [38]. In a study of Cafiso et al., 83.3% of CRA and TCP positive isolates were *ica* positive [34]. We also observed the presence of *icaAD* genes in many biofilm-negative strains in phenotypic assays, indicating the importance of genotypic assay in in vitro biofilm detection. However, evidences showing *ica* independent biofilm production suggest that *ica* negative results may not always reveal the absence of biofilm production. For instance; the presence of accumulation associated protein (*aap*) or Bap homolog protein (*bhp*) have been demonstrated to be responsible for biofilm production, suggesting the presence of PIA independent mechanisms in biofilm formation [37, 45–48].

### Conclusion

The present study demonstrated the causal link between the presence of *icaAD* genes and biofilm production in the clinical staphylococcal isolates. Although TCP method was found to be superior to other phenotypic assays in terms of specificity and sensitivity, it was not well correlated with the genotypic assay. Taken together, these results suggest the use of genotypic assay along with the TM method in routine diagnostics to detect biofilm producers in clinical samples.

## Limitations

Evaluation of biofilm production based merely on different nutrient supplements in vitro phenotypic assay may jeopardize the detections of biofilm production which depend on various factors. In addition, we examined presence of *ica* genes that are associated with PIA dependent biofilm production. This likely limits the detection of *ica* independent biofilm production.

## Additional files

**Additional file 1: Table S1.** Frequency of *Staphylococcal* spp. in different clinical sample.

**Additional file 2: Table S2.** Correlation of Biofilm Production with *ica* genes.

**Additional file 3: Table S3.** Biofilm detection among *S. aureus* and CoNS by different methods.

**Additional file 4: Table S4.** Statistical evaluation of different phenotypic methods compared with standard TCP method.

## Abbreviations

CoNS: Coagulase Negative *Staphylococcus aureus*
CRA: Congo-reg Agar
TM: tube method
TCP: tissue culture plate method
PCR: polymerase chain reaction
KIST: Kathmandu institute of science and technology
CVC: central venous catheter
ET: endotracheal tube
PIA: polysaccharide intercellular adhesion
PNAG: poly-*N*-acetylglucosamine
OD: optical density
°C: degree centigrade
*icaADBC*: intercellular adhesion operon containing *icaA, icaD, icaB* and *icaC* genes
*aap*: accumulation associated protein
*bhp*: Bap homolog protein

## References

[1] Donlan RM, Costerton JW (2002). Biofilms: survival mechanisms of clinically relevant microorganisms. Clin Microbiol Rev.

[2] Gotz F (2002). Staphylococcus and biofilms. Mol Microbiol.

[3] O’Gara JP (2007). ica and beyond: biofilm mechanisms and regulation in *Staphylococcus epidermidis* and *Staphylococcus aureus*. FEMS Microbiol Lett.

[4] Cerca N, Martins S, Cerca F, Jefferson KK, Pier GB, Oliveira R, Azeredo J (2005). Comparative assessment of antibiotic susceptibility of coagulase-negative staphylococci in biofilm versus planktonic culture as assessed by bacterial enumeration or rapid XTT colorimetry. J Antimicrob Chemother.

[5] Bazzoun DA, Harastani HH, Shehabi AA, Tokajian ST (2014). Molecular typing of *Staphylococcus aureus* collected from a Major Hospital in Amman, Jordan. J Infect Dev Ctries.

[6] Mack D, Fischer W, Krokotsch A, Leopold K, Hartmann R, Egge H, Laufs R (1996). The intercellular adhesin involved in biofilm accumulation of *Staphylococcus epidermidis* is a linear beta-1,6-linked glucosaminoglycan: purification and structural analysis. J Bacteriol.

[7] Oliveira A, Cunha Mde L (2010). Comparison of methods for the detection of biofilm production in coagulase-negative staphylococci. BMC Res Notes.

[8] Vuong C, Kocianova S, Voyich JM, Yao Y, Fischer ER, DeLeo FR, Otto M (2004). A crucial role for exopolysaccharide modification in bacterial biofilm formation, immune evasion, and virulence. J Biol Chem.

[9] Wojtyczka RDOK, Kępa M, Idzik D, Dziedzic A, Mularz T, Krawczyk M, Miklasińska M, Wąsik TJ (2014). Biofilm formation and antimicrobial susceptibility of *Staphylococcus epidermidis* strains from a hospital environment. Int J Environ Res Public Health.

[10] Dobnisky SK, Rohde H, Bartscht K, Knobloch J, Horskotte M, Dietrich M (2003). Glucose related dissociation between ica ADBC transcription and biofilm expression by *Staphylococcus epidermidis*: evidence for an additional factor required for polysaccharide intercellular adhesion synthesis. J Bacteriol.

[11] Tang Y-W (2010). SCW: *Staphylococcus aureus*: an old pathogen with new weapons. Clin Lab Med.

[12] Stewart PS, Costerton JW (2001). Antibiotic resistance of bacteria in biofilms. Lancet.

[13] Hamilton MA (2002). Testing antimicrobials against biofilm bacteria. J AOAC Int.

[14] von Eiff CPG, Heilmann C (2002). Pathogenesis of infections due to coagulase-negative staphylococci. Lancet Infect Dis.

[15] Klingenberg CAE, Rønnestad A, Sollid JE, Abrahamsen TG, Kjeldsen G, Flaegstad T (2005). Coagulase-negative staphylococcal sepsis in neonates. Association between antibiotic resistance, biofilm formation and the host inflammatory response. Pediatr Infect Dis J.

[16] Arciola CR, Campoccia D, Ravaioli S, Montanaro L (2015). Polysaccharide intercellular adhesin in biofilm: structural and regulatory aspects. Front Cell Infect Microbiol.

[17] Nathan K, Archer M, Mazaitis J, Costerton J, Powers M, Mark E (2011). *Staphylococcus aureus* biofilms. Virulence.

[18] Mathur TSS, Khan S, Upadhyay DJ, Fatma T, Rattan A (2006). Detection of biofilm formation among the clinical isolates of staphylococci: An evaluation of three different screening methods. Indian J Med Microbiol.

[19] Cheeseburg M (2006). District laboratory practice in tropical countries.

[20] Freeman DJ, Falkiner FR, Keane CT (1989). New method for detecting slime production by coagulase-negative staphylococci. J Clin Pathol.

[21] Christensen GD, Simpson WA, Yonger JJ, Baddor LM, Barrett FF, Melton DM, Beachey EH (1985). Adherence of coagulase-negative staphylococci to plastic tissue culture plates: a quantitative model for the adherence of staphylococci to medical devices. J Clin Microbiol.

[22] Cramton SE, Gerke C, Schnell NF, Nichols WW, Gotz F (1999). The intercellular adhesion (ica) locus is present in *Staphylococcus aureus* and is required for biofilm formation. Infect Immun.

[23] Atshan SS, Nor Shamsudin M, Sekawi Z, Lung LT, Hamat RA, Karunanidhi A, Mateg Ali A, Ghaznavi-Rad E, Ghasemzadeh-Moghaddam H, Chong Seng JS (2012). Prevalence of adhesion and regulation of biofilm-related genes in different clones of *Staphylococcus aureus*. J Biomed Biotechnol.

[24] Shrestha LB, Bhattarai NR, Khanal B (2017). Antibiotic resistance and biofilm formation among coagulase-negative staphylococci isolated from clinical samples at a tertiary care hospital of eastern Nepal. Antimicrob Resist infect Control.

[25] Molnar C, Hevessy Z, Rozgonyi F, Gemmell CG (1994). Pathogenicity and virulence of coagulase negative staphylococci in relation to adherence, hydrophobicity, and toxin production in vitro. J Clin Pathol.

[26] Otto M (2009). *Staphylococcus epidermidis*–the ‘accidental’ pathogen. Nat Rev Microbiol.

[27] Widerstrom M, Wistrom J, Sjostedt A, Monsen T (2012). Coagulase-negative staphylococci: update on the molecular epidemiology and clinical presentation, with a focus on *Staphylococcus epidermidis* and *Staphylococcus saprophyticus*. Eur J Clin Microbiol Infect Dis.

[28] Costerton JW, Stewart PS, Greenberg EP (1999). Bacterial biofilms: a common cause of persistent infections. Science.

[29] Arciola CR, Baldassarri L, Montanaro L (2001). Presence of icaA and icaD genes and slime production in a collection of staphylococcal strains from catheter-associated infections. J Clin Microbiol.

[30] Arciola CR, Collamati S, Donati E, Montanaro L (2001). A rapid PCR method for the detection of slime-producing strains of *Staphylococcus epidermidis* and *S. aureus* in periprosthesis infections. Diagn Mol Pathol..

[31] Nasr RA (2012). Biofilm formation and presence of icaAD gene in clinical isolates of staphylococci. Egypt J Med Human Genet.

[32] Namvar AE, Asghari B, Ezzatifar F, Azizi G, Lari AR (2013). Detection of the intercellular adhesion gene cluster (ica) in clinical *Staphylococcus aureus* isolates. GMS Hyg Infect Control..

[33] Cue D (2012). Genetic regulation of the intercellular adhesion locus in staphylococci. Front Cell Infect Microbiol.

[34] Cafiso V, Bertuccio T, Santagati M, Campanile F, Amicosante G, Perilli MG, Selan L, Artini M, Nicoletti G, Stefani S (2004). Presence of the ica operon in clinical isolates of *Staphylococcus epidermidis* and its role in biofilm production. Clin Microbiol Infect.

[35] Gad GF, El-Feky MA, El-Rehewy MS, Hassan MA, Abolella H, El-Baky RM (2009). Detection of icaA, icaD genes and biofilm production by *Staphylococcus aureus* and *Staphylococcus epidermidis* isolated from urinary tract catheterized patients. J Infect Dev Ctries.

[36] Ruzicka F, Hola V, Votava M, Tejkalova R, Horvat R, Heroldova M, Woznicova V (2004). Biofilm detection and the clinical significance of *Staphylococcus epidermidis* isolates. Folia Microbiol.

[37] Los R, Sawicki R, Juda M, Stankevic M, Rybojad P, Sawicki M, Malm A, Ginalska G (2010). A comparative analysis of phenotypic and genotypic methods for the determination of the biofilm-forming abilities of *Staphylococcus epidermidis*. FEMS Microbiol Lett.

[38] de Silva GD, Kantzanou M, Justice A, Massey RC, Wilkinson AR, Day NP, Peacock SJ (2002). The ica operon and biofilm production in coagulase-negative Staphylococci associated with carriage and disease in a neonatal intensive care unit. J Clin Microbiol.

[39] Arciola CR, Campoccia D, Gamberini S, Cervellati M, Donati E, Montanaro L (2002). Detection of slime production by means of an optimised Congo red agar plate test based on a colourimetric scale in *Staphylococcus epidermidis* clinical isolates genotyped for ica locus. Biomaterials.

[40] Fitzpatrick F, Humphreys H, O’Gara JP (2005). The genetics of staphylococcal biofilm formation–will a greater understanding of pathogenesis lead to better management of device-related infection?. Clin Microbiol Infect.

[41] Cho SH, Naber K, Hacker J, Ziebuhr W (2002). Detection of the icaADBC gene cluster and biofilm formation in *Staphylococcus epidermidis* isolates from catheter-related urinary tract infections. Int J Antimicrob Agents.

[42] Johannes KM (2002). Evaluation of different methods of biofilm formation in *Staphylococcus aureus*. Med Microbiol Immunol.

[43] Arciola CR, Campoccia D, Baldassarri L, Donati ME, Pirini V, Gamberini S, Montanaro L (2006). Detection of biofilm formation in *Staphylococcus epidermidis* from implant infections Comparison of a PCR-method that recognizes the presence of ica genes with two classic phenotypic methods. J Biomed Mater Res..

[44] Mack D, Rohde H, Dobinsky S, Riedewald J, Nedelmann M, Knobloch JK, Elsner HA, Feucht HH (2000). Identification of three essential regulatory gene loci governing expression of *Staphylococcus epidermidis* polysaccharide intercellular adhesin and biofilm formation. Infect Immun.

[45] Rohde H, Burandt EC, Siemssen N, Frommelt L, Burdelski C, Wurster S, Scherpe S, Davies AP, Harris LG, Horstkotte MA (2007). Polysaccharide intercellular adhesin or protein factors in biofilm accumulation of *Staphylococcus epidermidis* and *Staphylococcus aureus* isolated from prosthetic hip and knee joint infections. Biomaterials.

[46] McCann MT, Gilmore BF, Gorman SP (2008). *Staphylococcus epidermidis* device-related infections: pathogenesis and clinical management. J Pharm Pharmacol.

[47] Qin Z, Yang X, Yang L, Jiang J, Ou Y, Molin S, Qu D (2007). Formation and properties of in vitro biofilms of ica-negative *Staphylococcus epidermidis* clinical isolates. J Med Microbiol.

[48] Jiang J (2006). Influence of ica transcription on biofilm phenotype of *Staphylococcus epidermidis* clinical isolates. Shanghai Med J.

